# Quality of life and psychological status in people with acromegaly in relation to disease-related facial changes

**DOI:** 10.1530/EC-24-0545

**Published:** 2025-04-25

**Authors:** Maria Antonia Martínez-Momblán, Sergio Alonso-Fernández, Montserrat Marques-Pamies, Isabel Salinas, Federico Vázquez, Berta Soldevila, Diego Asensio-Wandosell, Raquel Ciriza, Alicia Santos, Elena Valassi, Susan M Webb, Manel Puig-Domingo

**Affiliations:** ^1^ Fundamental and Clinical Nursing Department, Nursing Faculty, University of Barcelona (UB), Feixa Llarga, L'Hospitalet de Llobregat, Barcelona, Spain; ^2^ CIBERER Unit 747, ISCIII, Madrid, Spain; ^3^ Department of Medicine, Univ Autònoma Barcelona, Barcelona, Spain; ^4^ GRIN Research Group, IDIBELL, Bellvitge Biomedical Research Institute, L'Hospitalet de Llobregat, Barcelona, Spain; ^5^ Endocrinology and Nutrition Service, Germans Trias i Pujol Hospital and Research Institute, Barcelona, Spain; ^6^ Department of Medicine, Autonomous University of Barcelona (UAB), Badalona, Barcelona, Spain; ^7^ Endocrinology and Nutrition Service, Hospital General de Granollers, Granollers, Barcelona, Spain; ^8^ Spanish Association of Acromegaly People, Barcelona, Spain; ^9^ Department of Endocrinology, Hospital S Pau, Barcelona, Spain; ^10^ Research Center for Pituitary Diseases, Institut de Recerca Sant Pau (IIB-Sant Pau), Barcelona, Spain; ^11^ Universitat Internacional de Catalunya, Barcelona, Spain

**Keywords:** acromegaly, facial changes, psychology, quality of life

## Abstract

The diagnosis of acromegaly is often delayed, leading to facial disfigurements that persist despite endocrine cure. We aimed to study the relationship between facial modifications, quality of life (QoL) and psychological status in patients with acromegaly.

**Methods:**

Sixty-five patients in remission or with hormonal disease control participated (29 women and 36 men; mean age 57.4 ± 13.5 years). The following variables were assessed: i) sex; ii) QoL by AcroQoL; iii) anxiety level with the STAI questionnaire; iv) self-esteem by the Rosenberg scale; and v) facial acromegaly phenotypical changes evaluated from face photographs by eight experienced endocrinologists using a Likert scale.

**Results:**

The global AcroQoL score for the whole cohort showed mid-range values, with higher median values for men (72.3 vs 56.6, *P* = 0.022) mostly dependent on the physical domain, which was higher for men (59.0 vs 34.9, *P* = 0.006), rather than the psychological domain (65.7 vs 54.5, *P* = 0.069). Anxiety scores were also higher for women (7.3 vs 5.9, *P* = 0.009). Self-esteem state was high (2.7 ± 0.6 for a maximal value of 3) for the whole cohort without sex differences. A higher difference in the global phenotypic facial acromegaly features score from baseline and post-diagnosis was associated with a lower value on QoL mostly in women (−14.38 95% CI −5.25 to −23.52; *P*-value = 0.005), with less effect in men (−6.31 95% CI 0.61 to −13.22; *P*-value = 0.070).

**Conclusion:**

In controlled acromegaly, QoL is relatively preserved in both genders, although men have higher general and physical domain scores and lower anxiety status. Facial changes intensity negatively impacted QoL mostly in women.

## Introduction

Acromegaly is a rare, chronic condition primarily caused by the overproduction of growth hormone (GH), usually due to a pituitary adenoma. It affects approximately 0.2–1.1 individuals per 100,000 annually, with a prevalence ranging from 2.8 to 13.7 cases per 100,000 in the general population (1). One of the hallmark features of acromegaly is the gradual alteration of physical appearance, particularly in the extremities and face. These facial changes are often so distinctive that they can lead to diagnosis when noticed by healthcare professionals (2, 3, 4).

The disease carries a substantial burden, impacting patients medically, psychologically and socially. It disrupts daily activities and is linked to complications affecting multiple systems, including metabolic, cardiovascular, respiratory and musculoskeletal functions (5, 6). Even after successful treatment and achieving endocrine remission, many patients continue to experience chronic morbidity, likely due to prolonged exposure to excessive GH before diagnosis. This persistent morbidity is thought to contribute to the significant psychological distress observed in individuals with acromegaly (7, 8, 9, 10, 11, 12, 13).

Despite the well-established physical and emotional challenges associated with acromegaly, research exploring the relationship between self-perceived bodily changes – particularly facial disfigurement – and psychological outcomes such as anxiety, self-esteem and quality of life (QoL) remains limited (14, 15). In 2002, Webb and Badia introduced the AcroQoL questionnaire, a validated tool specifically designed to measure QoL in acromegaly patients (16, 17, 18, 19). Subsequent studies have identified various factors influencing QoL scores, including age, disease duration, radiotherapy, gender, disease activity and the presence of psychological and physical comorbidities. Among these, facial changes have a profound effect on self-perception and warrant closer examination. Notably, women with acromegaly tend to experience greater negative impacts on appearance-related QoL compared to men, a pattern also seen in other chronic conditions (9, 20).

Early diagnosis could significantly reduce the health consequences of acromegaly; however, diagnostic delays remain a critical issue. On average, it is estimated that it takes 8 years for men and 10 years for women to receive a diagnosis since disease initiation (21, 22, 23). These delays exacerbate the physical and psychological toll of the disease, highlighting the urgent need for increased awareness and earlier detection.

Given the scarcity of research examining the connection between morphometric facial changes and their psychological and QoL implications in acromegaly, this study aims to explore these relationships in a cohort of Spanish patients.

## Methods

### Patients

A cohort study was carried out in Spain between September 2020 and February 2022. Patients with acromegaly, followed as outpatients at Germans Trias and Sant Pau hospitals in Barcelona (Spain), were invited to participate. The inclusion criteria were ≥18 years of age, regular outpatient follow-up and acromegaly either in remission after surgery or controlled under medical treatment (with normal GH and IGF-1) at last visit.

Sixty-five patients were accepted to participate. Recruitment was performed by telephone or during the regular scheduled visits at the outpatient clinic. Over the last 4 years of follow-up, all patients were in a stable and hormonally controlled situation or in remission following surgery, with two or more visits per year, if required. Diagnosis had been reached 10–21 years before study entry. Time to hormonal control or cure since diagnosis ranged from 1 to 4 years.

### Study variables

The study variables included:

**A) Sociodemographic features**: age, sex, educational level, working activity and marital status.

**B) Anthropometric characteristics**: weight, height, body mass index (BMI) and blood pressure.

**C) Variables related to acromegaly**: tumor size at diagnosis, comorbidities including pituitary insufficiency and its replacement treatment, chronic arthropathy, diabetes mellitus, hypertension, obesity, sleep-apnea and cancer; treatments performed (surgery, radiotherapy and medical treatment used), recurrence rate and duration of disease activity, as recalled by the patient since initial symptoms until disease control.

**D) Quality of life**: QoL was evaluated using AcroQoL, a disease-specific questionnaire for patients with acromegaly, which assesses a global score and two subdimensions: physical function (eight items) and psychological well-being (14 items). The psychological dimension is subdivided into two subdimensions, namely appearance and personal relationships, containing seven items each. Each of the 22 items of the questionnaire is answered on a 5-point Likert scale. The highest achievable score is 110 points (which corresponds to an AcroQoL score of 100), indicating an excellent QoL, while the lowest score is 22 (corresponding to 0). The scale developed in Spanish has been translated and validated into multiple languages (13).

**E) Anxiety Level**: anxiety was measured through the State–Trait Anxiety Inventory (STAI) questionnaire, which measures two dimensions of anxiety: state anxiety and trait anxiety. It consists of a 6-item reduced version of the original STAI questionnaire (24), and comprises six items per scale, with a minimum score of 0 (no anxiety) and a maximum of 18.

**F) Self-esteem status**: self-esteem status was evaluated using the Rosenberg self-esteem scale; this questionnaire explores personal self-esteem as the feelings of personal worth and self-respect. The questionnaire is composed by ten items, answered on a 5-point Likert scale. Scores between 30 and 40 points indicate high self-esteem, 26–29 points indicate average self-esteem and less than 25 points indicate low self-esteem. The scale has been translated and validated into Spanish. The internal consistency of the scale is between 0.76 and 0.87 (25).

**G) Global phenotypic facial acromegaly features**: they were evaluated by examining facial photographs provided by the participants by eight trained endocrinologists who scored them on a Likert scale from 0 to 10 according to the perceived magnitude of global facial changes related to acromegaly (0, no facial abnormalities; 1–3, suggestive changes; 3–6, substantial changes; 6–9, severe changes; and >9, very severe facial acromegaly phenotypic changes). Three different facial photographs were evaluated for each patient, one 10 years before diagnosis (pre-diagnosis), another around the diagnosis period (diagnosis) and another at current follow-up, when the disease was either under remission or controlled by medical treatment with normal circulating GH and IGF-1.

For the evaluation of facial changes, particular attention was paid to the modifications of the nose, the margin of the eyes, cheekbones, cheek volume and the margin of the nostrils; facial soft tissue changes were also considered – particularly swelling of the lips. As previous publications reported greater enlargement of nasofrontal angles and greater mentolabial angles for females, these sexually dimorphic characteristics were especially evaluated. The concordance between physician’s scoring was also assessed using the intraclass correlation index. Facial score changes over time for each patient was defined by subtracting the scores obtained at post- and pre-diagnostic time-points. During photograph scoring, the endocrinologists were blinded as to the corresponding follow-up time-points (namely pre-diagnosis, at diagnosis or current).

**H) Hormonal measurements**: these included the whole set of pituitary hormones, and GH and IGF1 for acromegaly diagnosis and follow-up. Serum GH was measured at each center by automated immunoassays, all calibrated against WHO IS 98/574, mostly by Liaison XL, Diasorin (Italy), and for serum IGF1 concentrations that were also measured calibrated against WHO NISBC 2stIS 02/254 by Liaison XL, Diasorin (Italy).

### Data collection and statistical analyses

All clinical variables were retrieved from the electronic clinical history of the hospital, which contains all the clinical, pharmacological and follow-up variables of the patients and were included in a data collection form using an anonymized code following the data protection policy of the institution, and the facial photos provided by all participants.

Study variables were described in tables for the entire sample and stratified by sex. Categorical variables were described by the frequency and percentage of each category. Continuous variables were described by the mean and standard deviation or by the median and interquartile range, depending on the distribution of the variables. Normality tests were determined by the Kolmogorov–Smirnov test. Qualitative variables are presented as frequencies and percentages. Inferential analysis was carried out based on the *t*-test or Mann–Whitney-U tests against normality. Correlations were determined by Pearson’s correlation coefficient.

Concordance among photograph evaluations was assessed using the intraclass correlation coefficient (ICC), considering a fixed set of physician judges. The impact of the global phenotypic facial acromegaly features on subjects’ QoL, anxiety level and self-esteem was analyzed using a linear regression model (Supplementary table 1 (see section on Supplementary materials given at the end of the article)). In these models, subjects’ status (e.g., QoL, anxiety or self-esteem) was the dependent variable and the difference on the global phenotypic facial acromegaly features score from baseline photograph was the independent variable. The models were adjusted by age, depression, BMI and sex, and the interaction between the change in features and sex was also evaluated.

The conditions of use of the models were validated and, whenever possible, confidence intervals at 95% were calculated. All analyses were performed with the statistical program R version 4.4.0 (2024-06-14) for windows.

The study was approved by the Clinical Ethics Committee of the Germans Trias Hospital (registry code PI-19-247). Written informed consent was obtained from all participants.

## Results

### Patient characteristics

The study cohort consisted of 65 acromegaly patients, of which 29 (44.6%) were women and 36 were men (53.4%); the mean age was 57.4 ± 13.5. The sociodemographic profiles regarding education, place of residence and marital status did not differ between genders. The mean BMI was 27.4 ± 4.54 kg/m^2^ (28.4 ± 4.21 vs 26.5 ± 5.01, respectively, in men and women). According to the diameter of the tumor at the time of diagnosis, there were 28 (43%) microadenomas (<10 mm) and 37 (57%) macroadenomas (>10 mm). Description of the cohort characteristics is presented in Table 1.

**Table 1:** Sociodemographic, anthropometric and clinical variables.

		Males	Females
Sex (*n*, %)		36 (55.4%)	29 (44.6%)
Age (mean ± SD) whole cohort			
Age (mean ± SD)		56.36 (11.6)	58.83 (15.7)
Marital status	Married	19 (76.0%)	13 (59.1%)
	Widower	0 (0.0%)	3 (13.6%)
	Single	5 (20.0%)	6 (27.3%)
	Divorced	1 (4.0%)	0 (0.0%)
Working status	Active	14 (38.8%)	8 (27.5%)
	Not working	0	3 (10.3%)
	Retired	13 (36.1%)	12 (41.3%)
	Disabled	1 (7.1%)	2 (16.7%)
Educational status	Primary	6 (28.6%)	9 (45.0%)
	High school	8 (38.1%)	5 (25.0%)
	University	7 (33.3%)	6 (30.0%)
Weight	Pre-diagnosis	91.2 (9.8)	69.5 (13.2)
	Post-diagnosis or at study entry	93.0 (18.6)	76.5 (17.7)
Height		177.4 (14.1)	163.7 (6.9)
BMI		28.4 (4.2)	26.5 (5.0)
**Clinical variables**			
Tumor size	Microadenoma	16 (50.0%)	12 (52.2%)
	Macroadenoma	16 (50.0%)	11 (47.8%)
Relapse	Yes	14 (45.2%)	5 (25.0%)
	No	17 (54.8%)	15 (75.0%)
Remission	Cured	19 (54.3%)	19 (65.5%)
	Active under control	16 (45.7%)	10 (34.5%)
Surgery	Yes	32 (91.4%)	21 (72.4%)
	No	3 (8.6%)	8 (27.6%)
Surgical modality	Transsphenoidal	29 (93.5%)	19 (100.0%)
	Transcranial	1 (3.2%)	0 (0.0%)
Second surgery	Yes	5 (15.2%)	3 (10.7%)
	No	28 (84.8%)	25 (89.3%)
Radiotherapy	Yes	7 (30.4%)	6 (40.0%)
	No	16 (69.6%)	9 (60.0%)
Diabetes		9 (25.7%)	8 (27.6%)
Hypertension		17 (48.6%)	13 (44.8%)
Dyslipidemia		9 (30.0%)	10 (40.0%)
Metabolic syndrome		1 (4.5%)	0 (0.0%)
Heart failure		1 (4.5%)	1 (6.3%)
Depression		5 (21.7%)	5 (29.4%)
Osteoporosis		3 (11.1%)	2 (8.7%)

BMI, body mass index.

Thirty-seven patients (58%) fulfilled complete remission criteria after surgery, while the remaining 28 were under medical treatment with normal hormonal parameters at the time of the study.

At enrollment, the number of patients who had undergone surgery, radiation therapy or had received treatment with somatostatin analogs or dopamine agonists were 50 (76.6%), 17 (26%), 34 (52.3%) and 11 (16.9%), respectively. Hypopituitarism of different degree was present in 34 patients (52.3%), most frequently cortisol and gonadal axis deficiencies. All patients with hormonal deficiencies were under stable replacement treatment.

### QoL, psychosocial outcomes and gender

The global AcroQoL score performed at the last follow-up visit for the whole cohort showed mid-range values, with higher median values for men (72.3 vs 56.6, *P* = 0.022, Table 2). AcroQoL physical domain showed higher scores for men (59.0 vs 34.9, *P* = 0.006, Table 2), while there were no sex differences in the psychological domain (65.7 vs 54.5, *P* = 0.06, Table 2).

**Table 2:** Psychosocial outcomes and gender.


Variable	Mean ± SD *n* = 65	Males *n* = 36 (53.4%)	Females *n* = 29 (46.6%)	*P*
**AcroQoL**				
AcroQoL scores: global	65.1 (21.4)	72.3 (18.5)	56.6 (21.9)	0.022*
Physical dimension	47.9 (28.3)	59.0 (26.6)	34.9 (25.0)	0.006*
Psychological dimension	60.5 (19.5)	65.7 (18.3)	54.5 (19.8)	0.069^†^
Appearance subdimension	46.9 (25.3)	53.8 (22.6)	38.8 (26.5)	0.070*
Personal relationship subdimension	64.6 (27.3)	70.0 (28.8)	58.3 (24.9)	n.s.
**Rosenberg scale**				
Self-esteem	2.7 (0.6)	2.7 (0.5)	2.6 (0.7)	n.s.
**STAI scale**				
Anxiety scale questionnaire (trait)	5.6 (2.0)	5.3 (2.0)	5.8 (2.1)	n.s.
Anxiety scale questionnaire (state)	6.5 (1.9)	5.9 (1.8)	7.3 (1.7)	0.009^†^

* *T*-test.

^†^ Mann–Whitney-U test; n.s., non-significant.

Regarding the Rosenberg scale, a relatively high self-esteem state for the whole cohort was observed, as the median was 2.7 ± 0.6 (maximal value 3), without gender differences.

Regarding anxiety, a relatively high level of anxiety was found in the whole cohort, as about 80% of the participants showed a high STAI scale score, namely 5.6 for STAI-trait and 6.5 for STAI-state. Women presented higher anxiety levels than males according to both STAI-trait and STAI-state; thus, anxiety scored 7.3 ± 1.7 as a state and 5.8 ± 2.1 as a trait and both were statistically higher than that in men (*P* = 0.009).

### Facial acromegaly phenotype evolution and gender

Facial acromegaly features were evaluated individually by eight endocrinologists, who scored the severity of these phenotypical facial modifications from 0 to 10 at three different time-points of the biographical evolution of each patient: before diagnosis, at diagnosis and after diagnosis. The reliability of the physicians’ aggregated scores, as measured by the ICC, was high, either for the pre-diagnosis (ICC 0.86 (95% CI 0.75–0.93)), diagnosis (ICC 0.90 (95% CI 0.86–0.95)) and the post-diagnosis (ICC 0.92 (95% CI 0.85–0.96)) photographs. This indicates good reliability in the physicians’ assessments at each time-point.

Scores obtained at these three time-points were 4.3 ± 1.5, 5.8 ± 1.7 and 6.4 ± 1.5 (out of a total maximum possible score of 10). Thus, facial changes increased from the pre-diagnostic to the diagnostic/post-diagnostic periods, but it is remarkable that all subjects presented identifiable acromegaly facial changes even 10 years before diagnosis. In addition, it is noteworthy that the magnitude of facial acromegaly features at diagnosis tended to be greater in men compared to women, with a mean score of 6.9 ± 1.4 vs 5.3 ± 1.4 in women, although statistical differences were only detected in the post-diagnostic period (*P* = 0.03) (see Table 3).

**Table 3:** Facial appearance scores and gender.


	Whole cohort mean ± SD *n* = 65	Males mean ± SD *n* = 36	Females mean ± SD *n* = 29	*P*
Pre-diagnosis	4.3 ± 1.5	4.7 ± 1.5	3.4 ± 1.3	n.s.
At diagnosis	5.8 ± 1.7	5.8 ± 1.9	5.6 ± 1.3	n.s.
Post-diagnosis	6.3 ± 1.5	6.9 ± 1.4	5.3 ± 1.4	0.03

### Correlation between psychosocial outcomes and facial acromegaly scores

**Figure 1:**
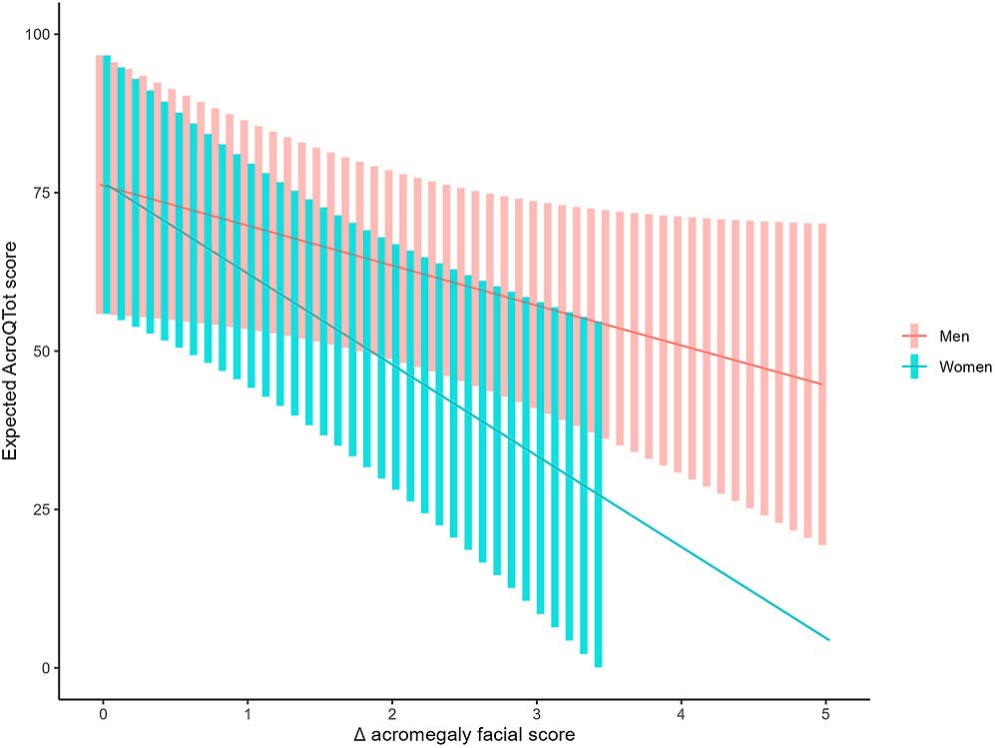
A higher difference in the global phenotypic facial acromegaly features score from baseline and post-diagnosis was associated with a lower value on QoL mostly in women (−14.38 95% CI −5.25 to −23.52; *P*-value = 0.005), although also in men, but without reaching statistical significance (−6.31 95% CI 0.61 to −13.22; *P*-value = 0.070).

**Figure 2:**
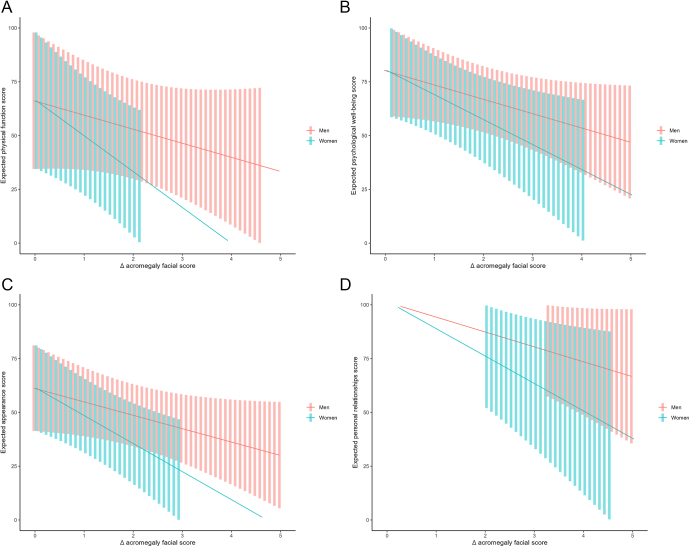
Differences observed between women and men in the different subdimensions of QoL; (A) physical function, (B) psychological well-being, (C) appearance and (D) personal relationships.

A significant effect was found on the difference in the global phenotypic facial acromegaly features score from baseline and post-diagnosis, and an interaction effect by sex. Thus, a higher difference in the global phenotypic facial acromegaly features score from baseline and post-diagnosis was associated with a lower value on QoL mostly in women (−14.38 (95% CI −5.25 to −23.52); *P*-value = 0.005), although also in men but without reaching statistical significance (−6.31 (95% CI 0.61 to −13.22); *P*-value = 0.070). Given a mean reduction of 2.3 points on acromegaly features score, the expected QoL score in men was 61.3 (95% CI 46.3–76.4) and in women 42.2 (95% CI 21.2–63.2), as shown in Fig. 1. These higher differences observed in women were present in all QoL dimensions compared to men: i) physical function: −16.68 95% CI −2.48 to −30.48; *P*-value = 0.025, and −6.54 95% CI 4.21 to −17.28; *P*-value = 0.211, men (Fig. 2A); ii) psychological well-being: in women −11.60 95% CI −2.16 to −21.04, *P* = 0.020, and −6.66 95% CI 0.48 to −13.81, *P* = 0.065 in men (Fig. 2B); iii) appearance: in women −13.04 95% CI −4.14 to −21.93, *P* = 0.007 and in men −6.23 95% CI 0.5 to −12.96; *P* = 0.067 (Fig. 2C); and iv) personal relationships: in women −12.76 95% CI −1.54 to −23.98, *P* = 0.029 and in men −6.92 95% CI 1.57 to −15.41; *P* = 0.102 (Fig. 2D).

In addition, a multivariate analysis to explore the impact of potential different confounders showed no influence of facial acromegaly scores and QoL scores: *P* = 0.56 when adjusting for age, *P* = 0.42 for BMI using nonparametric tests, *P* = 0.24 for educational level, *P* = 0.27 for medical treatment using Kruskal–Wallis, *P* = 0.68 for size of the adenoma, *P* = 0.91 for having been treated with radiotherapy and *P* = 0.71 for surgical treatment using Mann–Whitney test.

Finally, anxiety as measured by STAI trait and state questionnaires did not show statistical associations with the magnitude of facial changes, or with the individual scores obtained at each time-point.

## Discussion

The diagnosis of acromegaly is frequently delayed, resulting in persistent facial disfigurements that endure even after achieving endocrine remission. This study sought to explore the interplay between facial changes, QoL and psychological well-being in patients with acromegaly. Our findings revealed that acromegaly-related facial changes exert a persistent negative influence on psychological well-being, with women experiencing this effect more intensely than men. Our cohort showed mid-range global AcroQoL scores, with men reporting significantly higher scores than women, mostly in the physical domain and particularly in the appearance-related subdimension, where women tended to score lower. Anxiety levels were notably higher in women, although self-esteem was generally high across the cohort with no significant sex differences. Importantly, greater phenotypic facial changes from baseline to post-diagnosis were associated with lower QoL, particularly in women with a less pronounced effect in men. Thus, while QoL remains relatively preserved in both genders among patients with controlled acromegaly, men exhibit better overall and physical domain scores and lower anxiety levels.

Some studies have consistently demonstrated the existence of psychosocial alterations in people affected by acromegaly even after being in long-term remission. Greater emotional lability, a decreased QoL assessed by global AcroQoL scores, an increased anxiety state and a lower self-esteem have been detected in these patients by using appropriate exploratory tools (10, 12, 13, 14, 25).

The psychological disturbances of people suffering from acromegaly can be attributed to the general deleterious effects of a previous long non-diagnosed period and its associated comorbidities which impact the health status, which may persist also after disease remission or hormonal control. Facial disfigurement, especially after a long diagnostic delay, may be very striking, obviously contributing to the psychological burden in these patients (26). The loss of one’s own face image caused by acromegaly may theoretically lead to a tendency toward depression and social isolation, this latter as a reaction of self-protection from social stigmatization through less social exposure. Although the general AcroQoL scores for our cohort were not particularly low for the whole group, we found that the appearance domain of AcroQoL was affected, with women scoring worse than men, and this sexual dimorphism also occurred for other variables studied in the present study. However, the relationship of the magnitude of change of facial acromegaly phenotype over time and self-esteem just showed a statistical trend, thus suggesting a process of adaptation and acceptance of such changes by the patients. As facial changes due to active acromegaly take years to be established, non-diagnosed affected people may consider these face changes mostly attributable to aging, and somehow accept them, with a relatively low psychological impact.

In recent years, different studies have analyzed the morphometric facial changes developed in acromegalic patients, but in these reports, there is no mention to the relationship between the morphometric score and its potential psychosocial consequences (27, 28, 29), except for the study of Imran *et al.* (9). This latter study showed that those individuals with a high acromegaly morphometric score were more likely to have apathy, less satisfaction with personal appearance, poorer personal relationships, poorer QoL as measured by the total QoL scale and in the physical appearance and personal relationships subscales. Our results are in line with these findings.

The psychological impact of a diagnosis of acromegaly is undoubtedly high and the patient’s awareness of the irreversibility of facial bone changes produced by the disease can be hard to accept; long-term, self-esteem may recover, but it cannot be ruled out that it potentially contributes to the existence of an increased anxiety level, as we found. Women are, or may be, particularly prone to be psychologically impacted at the time of diagnosis and this effect may persist for years, sometimes never recovering from such a situation. In this regard, we also found that this negative perception was more important in women even more than 5 years after disease control when worse AcroQoL results were present compared to men. Likewise, anxiety levels in women were significantly higher than in men, as also previously reported by others (9, 30).

After disease remission or hormonal control, even if the facial changes affecting soft tissue may have improved in parallel to control of GH overexposure, the relationship between self-esteem and facial scores only showed a trend, suggesting a low impact and certain resilience in such individuals to the overall acromegaly condition, as also reported regarding self-esteem in other studies (29, 31). In this regard, it is also remarkable that the facial scores did not decrease in the post-diagnosis evaluation, which reinforces adaptation to such a condition. In our cohort, there were low arthropathy reported symptoms, which may also explain the general QoL and psychological parameters evolution assessed over time in our study.

Different authors (14, 25) have found that anxiety levels remain high throughout the different phases of the disease, with a higher impact in women, as we also observed. In the present study, however, we found greater acromegaly facial morphometric changes in men than in women, especially in the post-diagnosis phase, as also evidenced by different reports, which confirms that these morphometric modifications are quite irreversible and show a sexual dimorphism (9, 32, 33); however, anxiety levels were higher in women, suggesting that if facial changes impact negatively, men seem more resistant to this process than women. Moreover, as we found, other studies have also shown that facial morphometric changes remain altered 2 years after disease remission (34, 35), indicating that this condition may potentially impact over time in acromegaly. In the study by Imran *et al.*, a correlation between acromegaly physical changes and poor psychological outcomes was found, whereas no such correlation existed regarding treatment modalities, disease control status, radiotherapy, malignancy, initial or recent GH/IGF1 or secondary hormonal deficiencies.

Our results on the psychosocial condition of participants may have been modulated when not aggravated by concurrent comorbidities that may contribute to the negative psychological status of these patients, although in our series, and probably due to the limited number of cases, no statistical interaction was found with these conditions and the studied outcomes (10, 36). In other studies, such as the one by Biermasz *et al.* (10), over 35% of patients considered cured showed elevated anxiety and depression scores, as we found in our cohort.

Finally, and not without importance, the COVID pandemic may have influenced the results of our study as it was performed during this period. Lack of usual follow-up due to lockdown requirements may have negatively impacted on anxiety and the overall well-being of participants, as reported by different authors (37, 38, 39). Thus, we cannot rule out that the lockdown caused by the COVID-19 pandemic contributed to the increased state of anxiety in these patients, although we believe that this exceptional situation does not invalidate our results. The most important limitation of the present study is the relatively small sample of participants, as the study finally included 65 patients; however, similar studies like ours, such as the one by Imran *et al.* included 55 patients. In this regard, the main findings related to self-esteem and facial changes remained at the limit of significance, while other categories of QoL were not specifically affected by these phenotypic modifications. Relative limitations of the tests employed are that self-esteem was assessed using a general scale that was not solely focused on physical attributes, which could potentially mask a distorted self-perception of one’s physical appearance; and that physical alterations were assessed by medical professionals, as we found it challenging to directly address this issue with the patients. This may have influenced the results of the study, but it should be noted that the patients’ perspective was partially assessed through the AcroQoL test. On the other hand, strengths of this study were the good concordance obtained in the facial scoring and the reliability of the validated instruments used to explore psychosocial characteristics and QoL of the participants.

In conclusion, acromegaly facial changes show a trend to a negative impact in the psychosocial condition of these Spanish patients, with maximal expression at the time of diagnosis, although global QoL scores do not show striking modifications at follow-up; these disturbances persist over time despite hormonal disease remission and are of higher intensity in women.

## Supplementary materials



## Declaration of interest

There is no conflict of interest that could be perceived as prejudicing the impartiality of the work reported.

## Funding

This research was funded by the Instituto de Salud Carlos IIIhttps://doi.org/10.13039/501100004587 grant PMP15/00027, co-funded by the European Regional Development Fund - European Union; and PMP22/00021, funded by the European Union-NextGenerationEU, both to Manel Puig-Domingo.

## Author contribution statement

Maria-Antonia Martínez-Momblan and Manel Puig-Domingo designed the study. All authors contributed to the study conception and design. Material preparation, data collection and analysis were performed by Maria-Antonia Martínez-Momblan and Sergio Alonso-Fernández. The first draft of the manuscript was written by Maria-Antonia Martínez-Momblan and final version by Manel Puig-Domingo and Susan M Webb; all authors reviewed and approved the final manuscript. We thank Cristian Tebe from the Research support department of Germans Trias Research Institute for reviewing the statistical analyses.
