# Supplementary material for: Quality of life and psychological status in people with acromegaly in relation to disease-related facial changes
Source: Endocr Connect. 2025 Apr 25;14(5):e240545. doi: 10.1530/EC-24-0545 (PMC12053918; doi:10.1530/EC-24-0545)
Supplement: Supplementary file 1 [file supplementary_materials.pdf]

Suplementary table 1. Differences in the global phenotypic facial acromegaly features score from baseline and post diagnosis, and an interaction effect by sex

	AcroQoL questionnaire				AcroQoL questionnaire				AcroQoL questionnaire			
Predictors	*Estimates*	*std. Error*	*CI*	*p*	*Estimates*	std. Error	CI	p	Estimates	std. Error	CI	p
(Intercept)	83.64	7.24	68.48 – 98.80	<0.001	75.56	6.57	61.82 – 89.31	<0.001	68.18	13.95	38.04 – 98.32	<0.001
difacropp	-6.66	2.36	-11.60 – -1.72	0.011								
Sex [women]	-20.23	7.25	-35.42 – -5.05	0.012								
difacropp × Sex men					-4.03	2.48	-9.22 – 1.17	0.121	-6.31	3.20	-13.22 – 0.61	0.070
difacropp × Sex women					-11.31	3.25	-18.12 – -4.50	0.003	-14.38	4.23	-23.52 – -5.25	0.005
DEpre [Sí]									16.18	13.93	-13.91 – 46.27	0.266
Observations	22				22				17			
R^2 / R^2 adjusted	0.420 / 0.358				0.390 / 0.326				0.474 / 0.352			
AIC	189.724				190.811				151.867			

A clinically relevant effect was found on the difference in the global phenotypic facial acromegaly features score from baseline and post diagnosis, and an interaction effect by sex. A higher difference in the global phenotypic facial acromegaly features score from baseline and post diagnosis was associated with a lower value on quality of life in women (-14.38 95%CI -5.25 to -23.52; p-value=0.005) and men (-6.31 95%CI 0.61 to -13.22; p-value=0.070). Given a mean reduction of 2.3 points on acromegaly features score the expected quality of life score in men was 61.3 (95%CI 46.3 to 76.4), and in women 42.2 (95%CI 21.2 to 63.2).
